# Spontaneous Rupture, Disappearance, and Reaccumulation of a Rathke's Cleft Cyst

**DOI:** 10.1155/2011/549262

**Published:** 2011-09-29

**Authors:** Katrina Maniec, Joe C. Watson

**Affiliations:** ^1^Inova Regional Neurosurgery Service and Department of Neurosurgery, Virginia Commonwealth University School of Medicine, Inova Campus, 3300 Gallows Road, Falls Church, VA 22042, USA; ^2^Department of Neuroscience, Inova Regional Neurosurgery Service, Falls Church, VA 22042, USA

## Abstract

Rathke's cleft cysts (RCCs) are benign epithelium-lined intrasellar cysts containing mucoid material and are believed to originate from the remnants of Rathke's pouch. Most are asymptomatic but may cause symptoms secondary to compression of adjacent structures such as visual disturbances and endocrinopathies, especially hypopituitary. Furthermore, inflammation such as an aseptic meningitis syndrome may be associated with these tumors, presumably resulting from leakage of cyst material into the subarachnoid space. We present a unique case of spontaneous rupture and complete disappearance of a known sella-suprasellar cyst associated with a severe headache syndrome, followed by cyst reaccumulation requiring surgery. Although this phenomenon is well accepted, to our knowledge, this is the first report of the complete disappearance of a Rathke's cyst presenting with the classic syndrome. Furthermore, it was remarkable how quickly it recurred and became symptomatic, providing evidence that an “empty sella syndrome” may indeed need clinical follow-up.

## 1. Case Report

**Figure 1:**
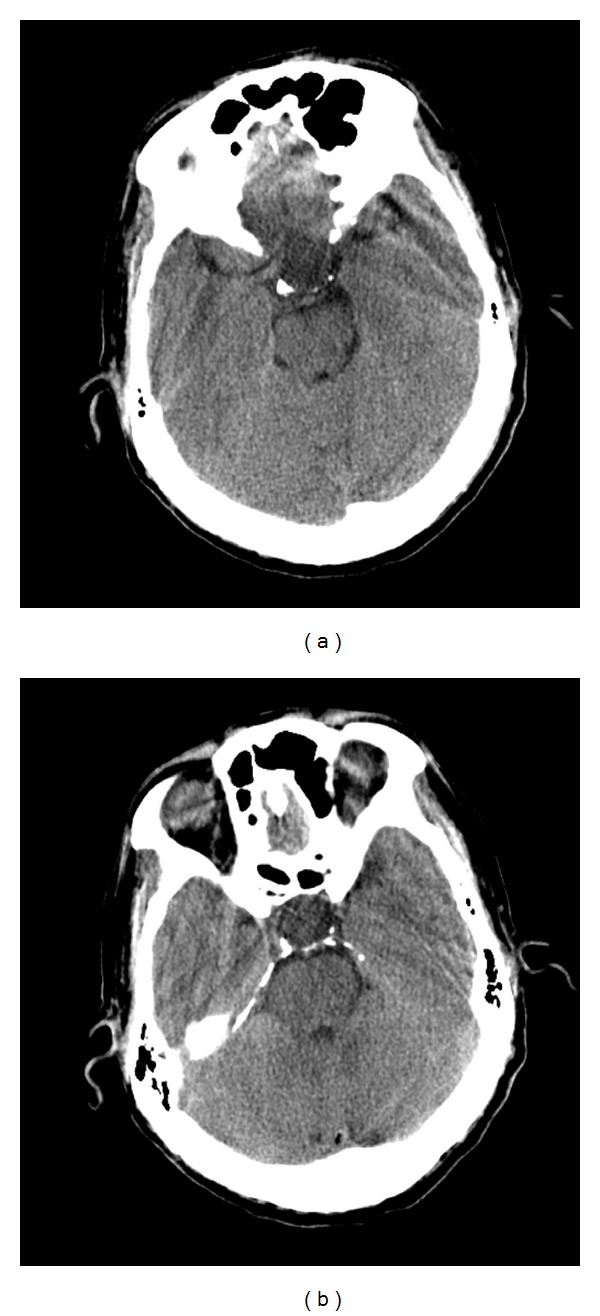
Noncontrast head CT in May revealing an enlarged sella turcica without evidence of calcifications, most consistent with a sella-suprasellar cystic mass.

**Figure 2:**
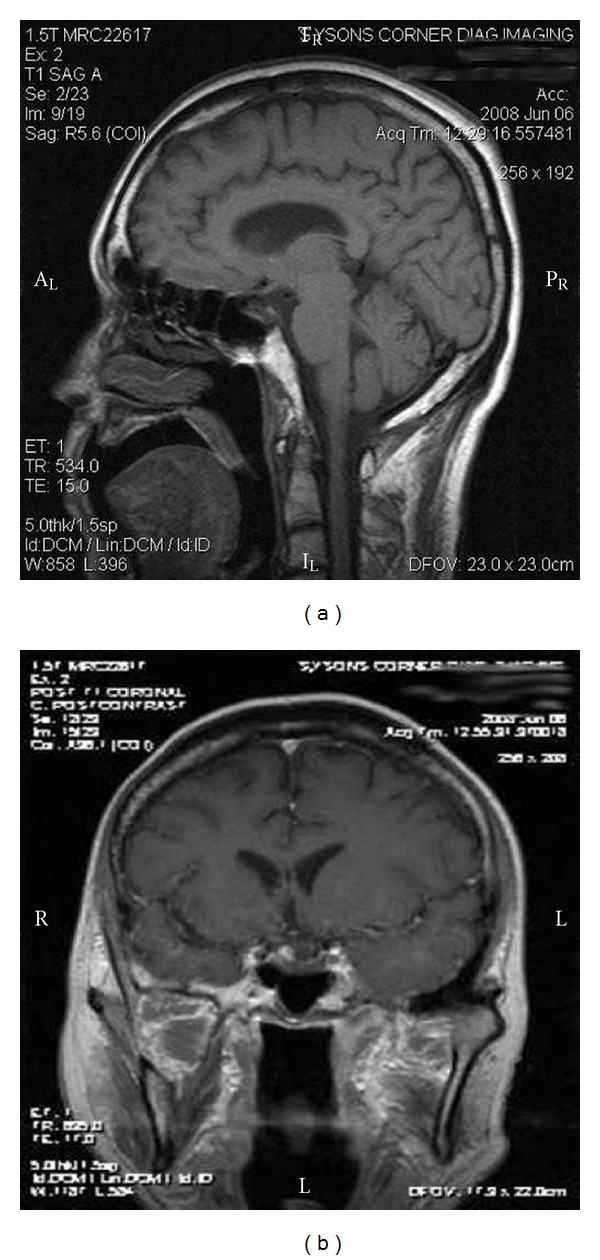
Sagittal and coronal postcontract MRI in early June revealing an empty sella without evidence of pituitary mass or cystic lesion.

**Figure 3:**
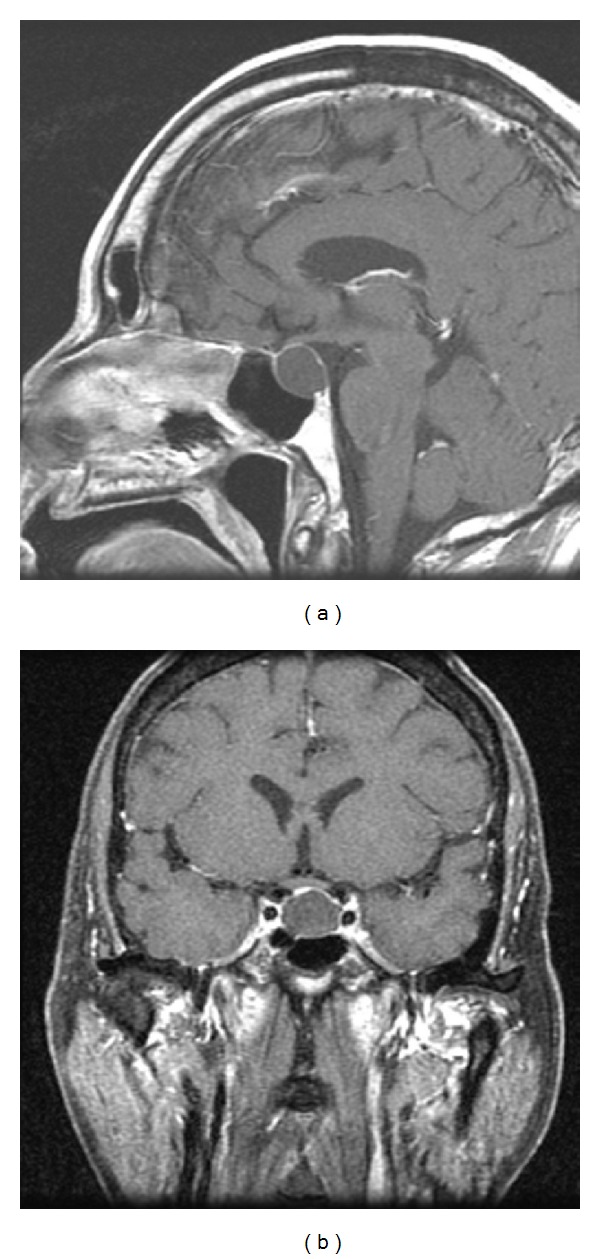
Sagittal and coronal postcontract MRI in late June revealing a sellar-suprasellar mass with some optic compression.

A 59-year-old-right handed man presented to the emergency department with a headache syndrome 6 months prior to admission. Head CT scan showed an enlarged sella and a suprasellar cystic mass (Figure 1). His headache resolved so he sought no further treatment until one month later (5 months prior to admission) at which time he complained of a severe headache associated with a stiff neck. Examination during this episode was documented to have a headache with photophobia and nuchal rigidity. Visual fields were full to confrontation. Once again his headache improved with acetaminophen. An MRI showed an empty sella (Figure 2). He re-presented to our office only three weeks later with a new MRI (Figure 3) that showed a large cystic lesion in the sella with suprasellar extension.

During this visit, he appeared thin but not cachectic. There were no stigmata of Cushing's or acromegaly. His pupils were equal, round, and reactive to light and the extra-ocular movements were full and normal.

Visual testing revealed a bitemporal upper quadrantanopia. Deep tendon reflexes were hypoactive throughout. The rest of the physical exam was within normal limits.

Lab results were consistent with hypopituitary: TSH = 1.37 IUI/mL, Free T4 = 0.70 ng/dL, AM Cortisol = 5 ug/dL, FSH = 4.2 mIU/mL, IGF-1 = 43 ng/mL, and Prolactin = 4.12 ng/mL.

He was started on thyroid replacement with levothyroxine and given the option of surgery. However, he was reluctant to agree to surgery and waited for 4 months. At this time he had developed a full bitemporal hemianopsia.

**Operative Findings.**

There was a very thin layer of capsule and gland over the cystic mass. Upon opening this cystic capsule, we encountered viscous, milky colored material consistent with Rathke's cyst contents. The cyst wall and fluid were sent to pathology and revealed ciliated epithelial cells consistent with a Rathke's cleft cyst.

Postoperatively, his visual complaints have been reversed and his fields are full to confrontation at 6 months. He remains on thyroid replacement.

## 2. Discussion

We describe and document radiographically a case of spontaneous rupture and subsequent reaccumulation of a Rathke's cleft cyst, a phenomenon thought possible but not previously shown. Rathke's cleft cysts (RCCs) may cause symptoms secondary to compression of adjacent structures such as visual disturbances and endocrinopathies, but have also been thought to cause an aseptic meningitis syndrome (e.g., Mollaret's meningitis), presumably resulting from leakage of cyst material into the subarachnoid space [1]. Ruptured Rathke's cysts are also associated with inflammatory hypophysitis of the pituitary [2–7]. We present a unique case of spontaneous rupture and complete radiographic disappearance of a known sella-suprasellar cyst associated with a severe headache syndrome, followed by cyst reaccumulation requiring surgery. Although the phenomenon of cyst leakage is well accepted, to our knowledge, this is the first report of complete disappearance of a Rathke's presenting with the classic syndrome. Such a spontaneous radiographic appearance has been described [8], but it was not proven histologically and the patient did not suffer from a meningitislike syndrome.

We were struck by the rapid radiographic reaccumulation of the cyst; he developed signs of optic compression and hypopituitarism within several months.

Clinicians are cautioned to be aware of this phenomenon when evaluating a patient with an empty sella. Treatment resulted in a radiographic remission of the Rathke's cyst, and relief of optic compression and visual loss, but failed to reverse the hypopituitary status as of a 12-month follow-up. It is presumed that the inflammatory effect on the gland was responsible.
